# Application of clinical and CT imaging features in the evaluation of disease progression in patients with COVID-19

**DOI:** 10.1186/s12890-023-02613-2

**Published:** 2023-09-06

**Authors:** Guobin Wu, Yunya Zhu, Xingting Qiu, Xiaoliang Yuan, Xiaojing Mi, Rong Zhou

**Affiliations:** 1 Respiratory and Critical Care Medicine, The First Affiliated Hospital of Gannan Medical University, No. 23 Qingnian Road, Zhanggong District, Ganzhou, 341000 Jiangxi China; 2 General Medicine, The First Affiliated Hospital of Gannan Medical University, No. 23 Qingnian Road, Zhanggong District, Ganzhou, 341000 Jiangxi China; 3 Radiology, The First Affiliated Hospital of Gannan Medical University, No. 23 Qingnian Road, Zhanggong District, Ganzhou, 341000 Jiangxi China

**Keywords:** CT imaging features, Clinical features, Progression, COVID-19

## Abstract

**Background:**

The Corona Virus Disease 2019(COVID-19) pandemic has strained healthcare systems worldwide, necessitating the early prediction of patients requiring critical care. This study aimed to analyze the laboratory examination indicators, CT features, and prognostic risk factors in COVID-19 patients.

**Methods:**

A retrospective study was conducted on 90 COVID-19 patients at the First Affiliated Hospital of Gannan Medical University between December 17, 2022, and March 17, 2023. Clinical data, laboratory examination results, and computed tomography (CT) imaging data were collected. Logistic multivariate regression analysis was performed to identify independent risk factors, and the predictive ability of each risk factor was assessed using the area under the receiver operating characteristic (ROC) curve.

**Results:**

Multivariate logistic regression analysis revealed that comorbid diabetes (odds ratio [OR] = 526.875, 95%CI = 1.384-1960.84, P = 0.053), lymphocyte count reduction (OR = 8.773, 95%CI = 1.432–53.584, P = 0.064), elevated D-dimer level (OR = 362.426, 95%CI = 1.228-984.995, P = 0.023), and involvement of five lung lobes (OR = 0.926, 95%CI = 0.026–0.686, P = 0.025) were risk factors for progression to severe COVID-19. ROC curve analysis showed the highest predictive value for 5 lung lobes (AUC = 0.782). Oxygen saturation was positively correlated with normally aerated lung volume and the proportion of normally aerated lung volume (P < 0.05).

**Conclusions:**

The study demonstrated that comorbid diabetes, lymphocyte count reduction, elevated D-dimer levels, and involvement of the five lung lobes are significant risk factors for severe COVID-19. In CT lung volume quantification, normal aerated lung volume and the proportion of normal aerated lung volume correlated with blood oxygen saturation.

## Introduction

Coronavirus Disease 2019 (COVID-19) can lead to severe respiratory complications, including acute respiratory failure, placing a substantial burden on healthcare systems worldwide to accommodate the influx of critically ill patients. For instance, patients with acute respiratory distress syndrome (ARDS) due to COVID-19 typically require intubation and intensive care unit (ICU) management, which is resource intensive [1]. Given the scarcity of mechanical ventilators and ICU care, it is crucial to accurately and promptly predict which COVID-19 patients require critical care. Moreover, early prognosis determination is beneficial when implementing new treatment approaches [2, 3]. Early detection and treatment of the disease are associated with reduced mortality in COVID-19 patients, particularly in those with severe conditions. Approximately 15% of COVID-19 patients develop ARDS, and over half of the patients in intensive care units experience hypoxia or respiratory failure [4]. Early prediction of severe disease progression is crucial, as it allows timely interventions that may improve the prognosis of critically ill COVID-19 patients [5]. Furthermore, knowing the progression of a patient’s illness enables appropriate allocation of scarce resources, such as mechanical ventilators or extracorporeal membrane oxygenation (ECMO) machines.

The diagnosis of COVID-19 requires the consideration of both SARS-CoV-2 nucleic acid test results and lung CT scan findings. However, the early stages of the disease are characterized by low positivity rates for SARS-CoV-2 nucleic acids and non-specific lung changes. Therefore, identifying early clinical indicators for the diagnosis of COVID-19 is currently a research hotspot [6]. Previous studies have shown that laboratory markers can provide reliable evidence for the precise treatment of COVID-19 [7]. Chest CT is the most sensitive radiological technique for diagnosing COVID-19 pneumonia and plays an irreplaceable role in early diagnosis and monitoring the clinical course of the disease, displaying diffuse lung changes from ground-glass opacities to consolidation and radiological changes at different stages of the disease course [8]. Chest CT not only provides clear positive indicators of COVID-19, substantially reducing the rate of missed diagnoses but also enables an overall assessment of the severity of the illness.

The high mortality rate of COVID-19 patients underlines the clinical significance of analyzing prognostic risk factors and adopting targeted treatment measures to improve patient prognosis and reduce mortality. At present, there are numerous reports on the factors influencing COVID-19 prognosis, but the results are not entirely consistent [9]. In COVID-19, all patients are at risk of progressing to severe illness, particularly those with comorbidities, such as obesity, cardiovascular disease, chronic pulmonary disease, hypertension, or cancer [10]. However, predicting when a COVID-19 patient will develop severe illness remains challenging. Based on this, the present study aimed to analyze laboratory examination indicators, CT features, and prognostic risk factors in COVID-19 patients.

## Methods

### Patient cohorts

We conducted a retrospective study of 90 patients included in this study were selected from a larger cohort of 350 diagnosed COVID-19 patients admitted to the First Affiliated Hospital of Gannan Medical University between December 17, 2022, and March 17, 2023. This selection period was chosen because it coincided with a surge in COVID-19 cases in our hospital and allowed us to accumulate enough patients for our retrospective study. The selection criteria were primarily based on the availability of complete data sets, including clinical history, laboratory test results, and serial chest CT scans, which were vital for our analysis. Patients were included if they had a confirmed epidemiological history, positive COVID-19 RT-PCR test results from respiratory or blood samples, at least one complete chest CT imaging examination, and complete clinical data and laboratory examination results. Patients with incomplete data or other viral pneumonia were excluded from our study. Clinical data, laboratory examination results, and imaging data were retrospectively collected using a hospital information system. Clinical data included sex, age, epidemiological history, underlying comorbidities, interval from onset to admission, hospital stay duration, clinical symptoms, treatment modalities, and prognosis. Laboratory examination results included white blood cell count, lymphocyte count, neutrophil-to-lymphocyte ratio (NLR), D-dimer, lactate dehydrogenase, C-reactive protein (CRP), and erythrocyte sedimentation rate (ESR). Imaging data mainly included serial chest CT images, as well as quantitative data such as lung aeration percentages and damaged lung volumes obtained through CT segmentation and reconstruction using the 3D-Slicer software.

### Inclusion criteria

(1) Patients with a definite epidemiological history and positive COVID-19 RT-PCR test results from respiratory or blood samples, (2) patients with at least one complete chest CT imaging examination, and (3) patients with complete clinical data and laboratory examination results.

### Exclusion criteria

1)Suspected patients with two consecutive negative COVID-19 nucleic acid tests from respiratory or blood samples, with an interval of over 24 h between tests; 2) patients without complete imaging examination, laboratory examination, and relevant clinical data; and 3) patients with other viral pneumonia, including severe acute respiratory syndrome (SARS), Influenza A virus, Influenza B virus, and Respiratory Syncytial Virus.

### Classification criteria

(1) Mild: Mild clinical symptoms with no radiographic evidence of pneumonia. (2) Moderate: Presence of fever and respiratory symptoms with radiographic evidence of pneumonia. (3) Severe: Adults meeting any of the following criteria:1) dyspnea with a respiratory rate (RR) ≥ 30 breaths/min; 2) oxygen saturation (SpO2) ≤ 93% at rest while breathing ambient air; 3) arterial partial pressure of oxygen (PaO2) to fraction of inspired oxygen (FiO2) ratio ≤ 300 mmHg (1 mmHg = 0.133 kPa); and 4) progressive worsening of clinical symptoms, with radiographic evidence of lesion progression > 50% within 24–48 h. (4) Critical: Meeting any of the following conditions:1) respiratory failure requiring mechanical ventilation; 2) shock; and 3) multiple organ failure necessitating intensive care unit (ICU) management. Based on these criteria, this study classified 30 mild and 26 moderate cases into the non-severe group (n = 56), and 28 severe and 6 critical cases into the severe group (n = 34).

### CT image analysis

**Fig. 1:**
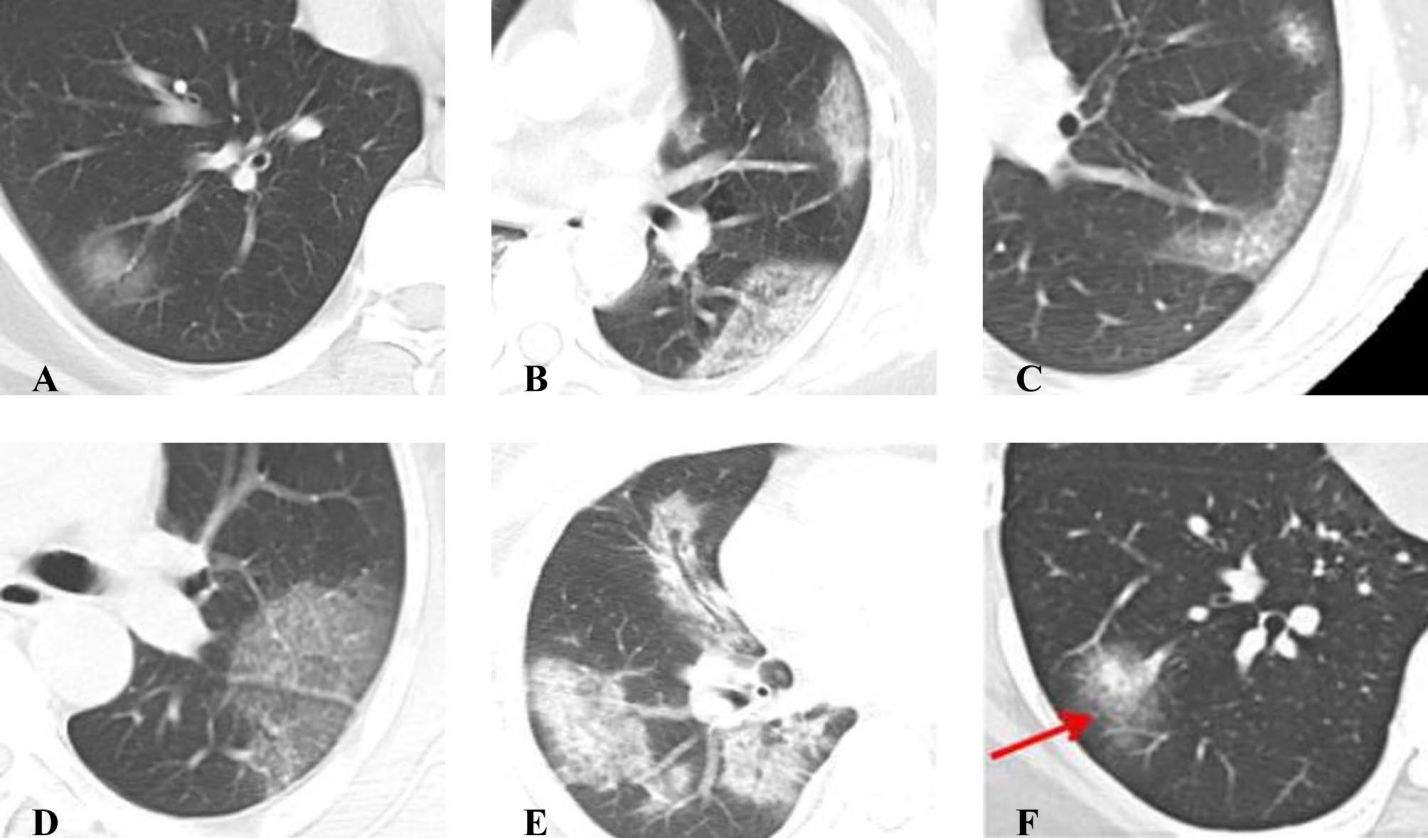
**A~ F**. Illustrations of Specific Signs: **(A)** Pure Ground-Glass Opacity (GGO); **(B)** Pleural Parallel Sign; **(C)** Vascular Shadowing; **(D)** Paving Stone Sign; **(E)** Bronchial Air Sign; **(F)** Halo Sign

Two experienced radiologists, specializing in chest imaging, independently and blindly reviewed the patients’ initial CT scans upon admission. The main evaluation criteria were as follows: (1) duration between the onset of symptoms and the CT scan; (2) number of lesions: no lesions, solitary lesions, or multiple lesions (≥ 2); (3) involvement of lung lobes: recording the lesion locations according to lung lobes and calculating the total number of affected lobes; (4) lesion distribution: peripheral (outer 1/3 of the lung fields), central, or central/peripheral diffuse distribution; and (5) lesion density: pure ground-glass opacity (GGO), GGO with partial consolidation or interlobular septal thickening, large reticular patterns (paving stone sign), or pure consolidation/fibrotic streaks (Fig. 1).

### Quantitative CT analysis

**Fig. 2:**
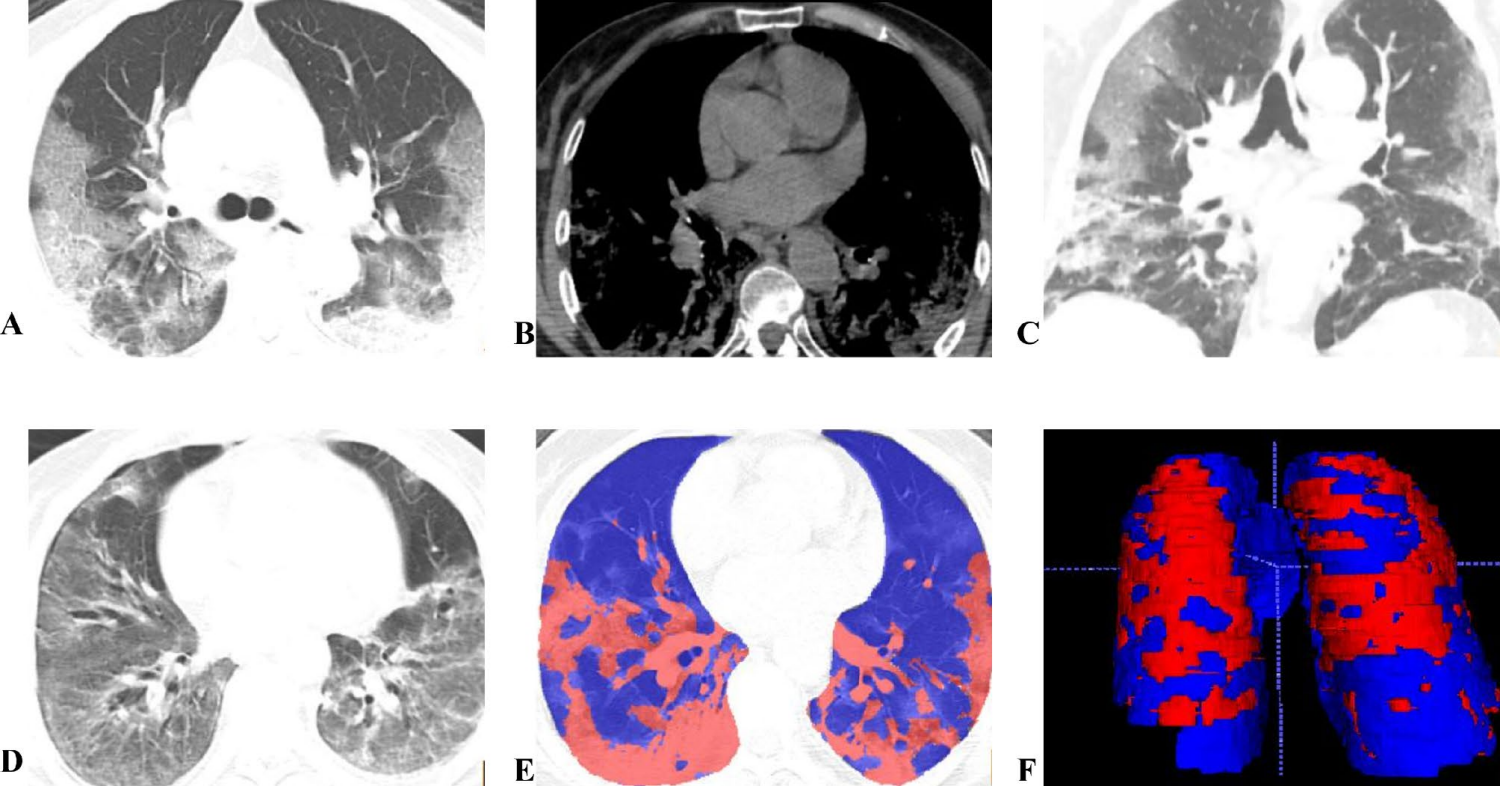
**A~ F**. Case Illustrations of Severe Group: Female, 71 years old, onset during close contact isolation observation. Figures A ~ C show chest CT one week after the first positive nucleic acid test, revealing diffuse GGO combined with consolidation in both lungs, a trend of merging in lobular distribution, and consolidation density mainly distributed along the pleura of both lower lobes, accompanied by the paving stone sign and parallel pleural sign. Figure D shows a follow-up CT 30 days after the onset, with diffuse GGO and consolidation in both lungs, bronchial dilatation, and fibrotic changes. Figures E, F utilize 3D Slicer for semi-automatic segmentation; the blue area represents Normally Aerated Lung tissue (NAL), and the red area represents poorly ventilated lung tissue (Compromised Lung, CL). In Figure F, the 3D volume shows widespread consolidated areas in both lungs, with NAL volume of 2207.5ml, and CL volume of 680.9ml. This patient suffered damage to over 30% of lung capacity, with concurrent respiratory failure and ARDS, requiring tracheal intubation treatment

All chest CT images of the cases were downloaded from the Picture Archiving and Communication System (PACS) in DICOM format, anonymized, and input into the medical image computation-specific segmentation suite (3D Slicer, www.slicer.org) [11] using semi-automatic segmentation algorithms (chest imaging platform, chest CT segmentation platform). First, automatic segmentation of lung volume was performed using the average density CT threshold method, excluding the main pulmonary arteries, bronchi, all mediastinal structures, and pleural effusions. Next, the lung volume was manually refined using 3D tools such as spherical brushes or erasers. The complete lung segments included two lungs with interstitial structures, segmental vessels, and bronchi. After calculating the total lung volume, the lung volumes were classified into normally aerated lung (NAL), compromised lung (CL), and hyperinflated lung (emphysema), based on different CT values. CT values of -950 to -650 HU were defined as normally aerated lung tissue, 100 to -650 HU as compromised lung, and − 1024 to -950 HU as emphysematous lung density [12]. Normal lung aeration volume/proportion and compromised lung volume/proportion were also obtained (Fig. 2).

### Statistical methods

Statistical analyses were performed using SPSS version 25.0. Data conforming to a normal distribution were presented as mean ± standard deviation and compared between groups using the t-test. Non-normally distributed continuous variables are presented as M (Q1, Q3), and group comparisons were conducted using the rank-sum test. Count data are presented as the number of cases (percentage) and compared using the Mann-Whitney U test or Fisher’s exact probability test. Statistical significance was set at p < 0.05. Factors with significant intergroup differences (P < 0.05) were included in the logistic multivariate regression analysis to identify relatively independent risk factors. The predictive ability of each independent risk factor was assessed using the area under the receiver operating characteristic (ROC) curve. Pearson’s correlation coefficient (Pearson’s r) was used to analyze the correlation between oxygen saturation and CT lung aeration proportion indices, with P < 0.05 considered statistically significant.

## Results

### Patient characteristics

In this study, 90 COVID-19 patients were included, with 47 males and 43 females, aged 43.37 ± 17.62 years. All patients tested positive for COVID-19 using RT-PCR before or upon admission. The average time from symptom onset to admission was 4 (3,6) days, and the average hospital stay was 12 (10,16) days. The clinical symptoms included fever (59.2%), cough (73%), sputum production (36%), hemoptysis (3%), chest pain (1.5%), chest tightness (11.4%), muscle aches (25.2%), and dizziness (11.5%). 32% had underlying conditions, including cardiovascular disease (13%), type 2 diabetes (11%), hyperlipidemia (6%), and asthma (1%). Complications occurred in 27% of the patients during treatment.

Of these 90 patients, 53 were classified as mild and 37 as severe. The average age of severe cases was significantly higher than mild cases (52.0 ± 14.73 vs. 36.84 ± 17.27 years, P < 0.001). No significant differences were found between the two groups in terms of sex, white blood cell count, neutrophil-to-lymphocyte ratio, platelet count, arterial blood carbon dioxide partial pressure, total bilirubin, alanine aminotransferase, and creatinine (all P > 0.05). The severe cases had a higher rate of comorbidities, with a significant difference in diabetes (24.3% vs. 1.8%, P < 0.001). Upon laboratory tests, severe patients had lower lymphocyte counts (1.36 ± 0.65 vs. 1.64 ± 0.70, P = 0.075), lower albumin (13.5% vs.15.1%, P = 0.0037), higher aspartate aminotransferase [40.13(25.6,34.7) vs. 20.5 (17.3,24.7), P = 0.087], higher D-dimer levels [10 (27) vs. 4 (7.5), P = 0.084], higher lactate dehydrogenase levels [10 (27) vs. 4 (7.5), P = 0.006], elevated erythrocyte sedimentation rate [21 (76.4) vs. 12 (22.6), P < 0.001], fewer elevated C-reactive protein levels [0 vs. 10 (18.7), P < 0.001], and lower arterial blood oxygen partial pressure (76.00 ± 21.07 vs. 85.48 ± 22.35, P = 0.075) (Table 1).

**Table 1:** comparison of clinical data and laboratory examination results between non-severe group and severe group

Variable	Normal group (n = 53)	Severe group (n = 37)	Test value	P value
Age (years, $\bar x \pm s$)	36.84 ± 17.27	52.0 ± 14.73	4.852	<0.001
Gender [cases (%)]			0.337	0.745
Male	28(52.8)	19(51.4)		
Female	25(47.2)	18(48.6)		
Complicated with underlying diseases [number of cases (%)]	8	21		
Cardiovascular diseases	5(9.4)	7(18.9)	1.221	0.23
Type 2 diabetes	1(1.8)	9(24.3)	3.272	<0.001
Hyperlipidemia	1(1.8)	5(13.5)	1.482	0.157
Allergic asthma	1(1.8)	0	0.757	0.478
White blood cell [× 10^9^ beat L, $\bar x \pm s$]	4.98 ± 2.13	4.87 ± 1.26	1.042	0.254
Leukopenia [case number (%)]	8(15.1)	5(13.5)	0.547	0.574
Lymphocytes [× 10^9^ beat L, $\bar x \pm s$]	1.64 ± 0.70	1.36 ± 0.65	2.432	0.075
Lymphocytopenia [case number (%)]	17(32)	21(56.75)	2.157	0.037
NLR < 1 [number of cases (%)]	6(11.3)	2(5.4)	0.827	0.496
Platelets [× 10^9^ pound L, $\bar x \pm s$]	173.43 ± 45.22	195.34 ± 67.13	0.72	0.456
D-dimer elevation	4(7.5)	10(27.0)	2.746	0.084
Abnormal lactate dehydrogenase	4(7.5)	10(27.0)	2.785	0.037
ESR > 20 mm/L [number of cases (%)]	12(22.6)	21(76.4)	3.34	<0.001
CRP > 6 mg/L [number of cases (%)]	10(18.7)	0	4.947	<0.001
PaO2(mmHg,c $\bar x \pm s$)	85.48 ± 22.35	76.00 ± 21.07	2.586	0.075
PCO2(mmHg,$\bar x \pm s$)	41.92 ± 5.24	42.46 ± 6.64	0.536	0.536
Albumin (gmax L, albumin $\bar x \pm s$)	42.56 ± 3.67	38.83 ± 5.37	4.198	<0.001
Total bilirubin (μ mol/L, $\bar x \pm s$)	12.97 ± 7.19	15.89 ± 7.32	0.035	0.987
ALT[U/L,M(Q1,Q3)]	22.1(11.35, 25.23)	40.8(18.3, 35.4)	2.078	0.037
AST[U/L,M(Q1,Q3)]	20.5(17.3, 24.7)	40.13(25.6, 34.7)	3.136	0.087
Creatinine (μ mol/L, $\bar x \pm s$)	67.14 ± 18.19	68.78 ± 11.30	0.387	0.798

Note: NLR: neutrophil-lymphocyte ratio; ESR: erythrocyte sedimentation rate; CRP:C- reactive protein; PaO2: partial pressure of oxygen in arterial blood; PCO2: partial pressure of carbon dioxide in arterial blood; ALT: glutamic pyruvic transaminase; AST: glutamic oxaloacetic transaminase; leukopenia: leukopenia: WBC count less than 1.1 × 10^9^; lymphocytopenia: lymphocyte count < 1.5 × 10^9^/L

### Comparison of chest CT features and quantitative CT analysis

The patients’ initial HRCT upon admission was compared between the mild and severe groups. The average time from disease onset to CT scan was similar (5.72 ± 3.56/5.37 ± 3.87 days, P = 0.773). Both groups exhibited similarities in the lesion number, affected lung lobes, and lesion distribution. The mild group had more cases with no significant CT changes [21(39.6)/1 (2.7]), while the severe group presented with a higher proportion of multiple disseminated lesions [19(35.8)/27(72.9] ), with statistically significant differences (P < 0.001). The predominant lesion presentations were ground-glass opacity (GGO), GGO with partial consolidation or interlobular septal thickening, large reticular changes (paving stone sign), and consolidation/fibrous cord shadow. Both groups had a higher number of cases with GGO accompanied by partial consolidation/interlobular septal thickening [20(37.7)/ 26(70.2] ). The severe group had a significantly higher number of cases with three other lesion types than the mild group (P < 0.05). The lesions were predominantly distributed in the peripheral zones of both lungs (outer 1/3 of the lung field). Some patients exhibited a spread from the periphery to the central area, with a higher proportion in the severe group [4 (7.5)/17 (45.9), P < 0.001). The lesions mainly occurred in the bilateral lower lobes, including 22 cases of right upper lobe involvement [7(13.2)/15(50.5]), 17 cases of left middle lobe involvement [5(13.2)/12(32.4]), and 42 cases of right lower lobe involvement [18(34.0)/24(64.8]). The severe group had more cases with multiple affected lobes, including those involving all five lobes [3 (5.6)/10 (11]), with a statistically significant difference (P = 0.002) (Table 2).

**Table 2:** comparison of chest HRCT characteristics between two groups of patients on admission

Variable	Normal group (n = 44)	Severe group (n = 31)	Test value	P value
The time between CT scan and onset (d, $\bar x \pm s$)	4.8 ± 3.37	5.28 ± 3.50	0.345	0.734
Number of lesions			4.346	<0.001
None	21(39.6)	1(2.7)	4.085	<0.001
One focus [number of cases (%)]	4(7.5)	1(2.7)	0.847	0.346
Multiple lesions (≥ 2) [number of cases (%)]	19(35.8)	27(72.9)	4.263	<0.001
Involve the lobe of the lung	3(5.6)	10(11)	3.568	0.001
Upper lobe of right lung [number of cases (%)]	7(13.2)	15(50.5)	3.376	
Middle lobe of right lung [number of cases (%)]	5(13.2)	12(32.4)	3.088	0.002
Lower lobe of right lung [number of cases (%)]	18(34.0)	24(64.8)	3.564	<0.001
Focus distribution			3.146	0.001
Peripheral dominant [number of cases (%)]	19(35.8)	11(27.9)	0.486	0.645
Central / peripheral diffuse distribution [number of cases (%)]	4(7.5)	17(45.9)	4.643	<0.001
Density				
Pure GGO [number of cases (%)]	12(22.6)	14(37.8)	1.787	0.086
GGO with consolidation/ lobular interstitial hyperplasia [cases (%)]	20(37.7)	26(70.2)	3.854	<0.001
Large grid [number of cases (%)]	9(17.0)	19(51.3)	3.86	<0.001
Pure consolidation / fiber strand [number of cases (%)]	4(7.5)	10(27)	2.747	0.068
Number of pulmonary lobes involved			4.324	<0.001
1 [number of cases (%)]	6(11.3)	4(10.8)	0.092	0.953
2 [number of cases (%)]	6(11.3)	6(16.2)	0.687	0.375
3[number of cases (%)]	8(15.0)	6(16.2)	0.732	0.786
4 [number of cases (%)]	1(1.8)	2(5.4)	1.576	0.383
5 [number of cases (%)]	3(5.6)	10(27)	3.347	0.002
Total lung volume (ml, $\bar x \pm s$)	3458.1 ± 937.9	3458.5 ± 1436.7	3.956	0.19
NAL(ml, $\bar x \pm s$)	3745.4 ± 754.3	2362.2 ± 946.5	5.845	<0.001
CL(ml, $\bar x \pm s$)	136.2 ± 148.7	336.5 ± 227.2	4.363	<0.001
Volume of emphysema (ml, $\bar x \pm s$)	84.7 ± 52.3	93.6 ± 62.2	0.970	0.341
CLP (%)	3.1 ± 4.5	9.3 ± 11.4	3.132	<0.001

Note: GGO: ground-glass opacit; NAL: Normally aerated lung; CL: Compromised lung; CLP: compromised lung percentage of the total

### Risk factors for progression to severe COVID-19

Multivariate logistic regression analysis of clinical and CT data revealed that comorbid diabetes (OR = 526.875, 95%CI = 1.384-1960.84, P = 0.053), lymphocyte count reduction (OR = 8.773, 95%CI = 1.432–53.584, P = 0.064), elevated D-dimer level (OR = 362.426, 95%CI = 1.228-984.995, P = 0.023), and involvement of the five lung lobes (OR = 0.926, 95%CI = 0.026–0.686, P = 0.025) were risk factors for progression to severe COVID-19 (Table 3).

**Table 3:** Logistic regression analysis for severe patients

Variable	OR value	95%CI	P value
Age	0.943	(0.927,1.037)	0.924
Complicated with diabetes mellitus	526.875	(1.384,196.084)	0.053
Lymphocyte decrease	8.773	(1.432,53.584)	0.064
D-dimer elevation	362.426	(1.228,984.995)	0.023
Abnormal lactate dehydrogenase	0.963	(0.027,41.797)	0.975
ESR>20 mm/L	1.927	(0.136,19.886)	9.532
CRP>6 mg/L	39.126	(0.842,1753.668)	0.084
PaO2	0.972	(0.936,1.085)	0.686
Albumen	1.026	(0.625,1.897)	0.032
AST(U/L)	1.073	(0.936,1.197)	0.86
Number of lesions	18.173	(0.524,77.987)	0.984
Number of lung lobes involved = 5	0.926	(0.026,0.686)	0.025
Focus distribution	20.483	(0.737,57.189)	0.086
NAL(ml)	0.927	(0.927,0.997)	0.008
CL(ml)	1.075	(1.025,1.097)	0.035
CLP	1.037	(1.025,4.986)	0.084

Note: ESR: Erythrocyte Sedimentation Rate; CRP:C- reactive protein; PaO2: partial pressure of arterial oxygen; AST: aspartate aminotransferase; Lymphocyte decrease: lymphocyte count < 1.5 × 10^9^ / L; NAL: Normally aerated lung; CL: Compromised lung; CLP: compromised lung percentage

### ROC curve analysis

The predictive ability of the statistically significant results from multivariate regression analysis was assessed. The AUC for comorbid diabetes, lymphocyte count reduction, elevated D-dimer, and involvement of the five lung lobes were 0.673, 0.671, 0.683, 0.727, and 0.782, respectively, with the highest predictive value for 5-lobe involvement. High specificity was found for comorbid diabetes and elevated D-dimer levels, while high sensitivity was observed for 5-lobe involvement and reduced normal aerated lung tissue volume (Table 4).

**Table 4:** Analysis of receiver operator characteristic curve

Variable	AUC	Cut-off values	Sensitivity	Specificity	95%CI
Complicated with diabetes mellitus	0.673	0.084	15.75	88.94	(0.443,0.773)
Lymphocyte decrease	0.671	0.227	68.73	58.98	(0.526,0.762)
D-dimer increased	0.683	0.283	34.72	91.67	(0.572,0.772)
Number of lung lobes involved = 5	0.727	0.436	96.43	47.91	(0.616,0.874)
NAL	0.782	0.772	90.682	43.91	(0.726,0.773)

Note: Lymphocyte decrease: lymphocyte count < 1.5 × 10^9^ / L; NAL: Normally aerated lung

### Pulmonary lesion volume alterations in relation to time progression

The 39 severe cases had a total of 126 CT images, with a more rapid progression of pulmonary lesion volume compared with the mild group, reaching a peak at 11 days from disease onset. The 53 mild cases had a total of 159 CT images, with the disease mostly controlled within one week after admission, followed by a pneumonia absorption phase, with the pulmonary lesion volume peaking at 13 days post-onset and then entering the absorption phase.

### Correlation analysis between oxygen saturation and CT quantitative parameters

Oxygen saturation was positively correlated with normal aerated lung volume and its proportion (P < 0.05, correlation coefficients 0.626 and 0.516, respectively), but not with the volume or proportion of lung tissue affected by COVID-19 (Table 5).

**Table 5:** correlation analysis between oxygen saturation and CT quantitative parameters

Variable	NAL	CL	NALP	CLP
PaO2	0.626	-0.462	0.516	-0.416
P value	<0.001	0.062	0.026	0.036

Note: NAL: Normally aerated lung; CL: Compromised lung; NALP: Normally aerated lung percentage; CLP: compromised lung percentage

## Discussion

Lymphopenia is a prominent feature of critically ill COVID-19 patients owing to the targeted invasion of SARS-CoV viral particles, which disrupt the cytoplasmic components of lymphocytes, leading to their destruction [13, 14]. Results from deceased and severely ill patients indicate that the percentage of lymphocytes in the blood is negatively correlated with the severity and prognosis of COVID-19. Studies have shown that 10–12 days post-symptom onset, the lymphocyte percentage in patients with mild COVID-19 remains above 20%. In contrast, severe cases exhibited lymphocyte percentages < 20%. Patients who progress to severe and critical conditions often have comorbid diabetes, a significant decrease in PaO2, elevated lactate dehydrogenase and creatine kinase levels associated with myocardial enzyme spectrum abnormalities, and liver function changes with elevated AST and ALT levels. The results of this study are consistent with these findings. Elevated D-dimer levels in patients with severe COVID-19 suggest hyperfibrinolysis. Approximately 50% of COVID-19 patients exhibit elevated D-dimer levels, and the degree of elevation in fibrin degradation products (FDP) and D-dimer levels is significantly higher in severe and deceased patients than in mild and surviving patients. Comparing laboratory indices between survivors and deceased patients, deceased patients demonstrated consistently elevated D-dimer levels, which subsequently remained high [15, 16]. Although CRP levels should be elevated in COVID-19 patients due to excessive inflammatory responses and heightened immune reactions during the progressive stage, this study observed fewer cases with elevated CRP, potentially attributable to the limited number of cases and early hospitalization of some severely ill patients.

Chest CT is highly sensitive in detecting early disease, evaluating lesion characteristics and extent, and identifying subtle changes that chest X-rays may not detect. Typical chest CT manifestations of COVID-19 include multifocal, peripheral, bilateral, patchy, subsegmental, or segmental ground-glass opacities (GGO) and consolidations, often distributed along the bronchovascular bundles and subpleural spaces. Large GGOs accompanied by interlobular septal thickening may present with “paving stone signs " and other typical changes [17, 18]. Comprehensive analysis of imaging and clinical data in this study revealed that comorbid diabetes, chest CT lesions involving all five lung lobes at admission, decreased lymphocytes, and elevated D-dimer levels are relatively independent risk factors for progression to severe conditions. Previous research has suggested that lesions distributed along the periphery, a maximum lesion range > 10 cm, involvement of all five lung lobes, absence of pleural effusion, and enlargement of hilar and mediastinal lymph nodes are risk factors for high-risk stratification of COVID-19 patients [19]. In this study, severe cases exhibited more lesion counts, involvement of all five lung lobes, and a peripheral-to-central diffusion trend in their initial CT scans than mild cases, consistent with previous research. Major risk factors for acute respiratory distress syndrome (ARDS) and mortality in COVID-19 patients include advanced age, neutrophilia, and organ and coagulation dysfunction (e.g., elevated lactate dehydrogenase and D-dimer levels) [16]. There is a close relationship between the severity of clinical and imaging manifestations in COVID-19 patients. In this study, patients with CT findings of lesions involving all five lung lobes and lower normal lung aeration volumes were particularly noteworthy for their potential progression to critical condition. The number and volume of lung lesions are directly related to lung injury and function, with increased lung damage leading to reduced lung capacity, insufficient oxygen reserves, and subsequently decreased blood oxygen levels or respiratory failure, necessitating enhanced clinical management. As the extent and degree of COVID-19 lung damage increases, along with the accumulated range of affected lung tissue, patients’ respiratory function may be impacted, potentially manifesting as hypoxic states. Previous research has suggested that reduced lung function is significantly associated with the PaO2/FiO2 ratio and aggravated pulmonary dysfunction, and that refractory hypoxemia primarily results from intrapulmonary shunting, which occurs in poorly aerated and non-aerated lung regions [14]. In this study, CT findings showed a positive correlation between oxygen saturation and normal aerated lung volume and the proportion of normally aerated lung volume; however, the correlation was not very strong, and there was no correlation with the volume and proportion of damaged lung tissue affected by the virus.

Aksu et al. [20] examined splenomegaly in COVID-19 patients and compared lung involvement patterns and segmental lung infiltration with Total Lung Severity Score (TLSS) in patients with and without splenomegaly. Splenomegaly in COVID-19 pneumonia patients may cause consolidation, crazy pavement pattern, pleural band formations, interlobular septal thickening, and secondary TB sequelae, according to their study. TLSS was higher in splenomegaly patients and most often affected the superior right lower lobe. Splenomegaly may indicate more severe lung involvement in COVID-19 individuals. We did not examine splenomegaly in our patient group; however, our work underscores the importance of CT imaging characteristics and laboratory markers in COVID-19 disease progression evaluation. Comorbid diabetes, chest CT lesions in all five lung lobes upon admission, reduced lymphocytes, and higher D-dimer levels were independent risk factors for severe diseases. Our CT data also demonstrated a favorable association between oxygen saturation and normal aerated lung capacity, supporting Aksu et al. [21] emphasis on lung imaging in COVID-19 severity assessment. Whether splenomegaly is a risk factor in our context is of considerable interest and might be investigated further. Aksu et al. examined the NLR and PLR in COVID-19 pneumonia patients. COVID-19 pneumonia patients had considerably higher NLR and PLR than those without. They found a favorable connection between NLR and PLR and CT scan Total Lung Severity Score (TLSS). They advised using NLR and PLR as inflammatory markers to assess lung involvement and disease severity in COVID-19 patients. Instead of just lymphocyte count decline, our study examines several additional significant risk factors. These include diabetes, increased D-dimer levels, and CT scans showing all five lung lobes. A multivariate logistic regression study showed these risk factors as independent for severe COVID-19. Our study also used CT measurement to determine that blood oxygen saturation is connected with lung volume and proportion. This multi-element method to COVID-19 severity assessment is more comprehensive. Both Aksu et al. and our study emphasize the necessity of CT scan in COVID-19 severity assessment. However, their biomarkers and risk factors differ. Both studies show that a combination of laboratory examination markers and clinical features is needed to quantify COVID-19 severity beyond routine pulmonary imaging.

This study has integrated laboratory testing indicators, clinical features, and CT scan characteristics to form a comprehensive severity assessment system. While previous studies have focused on these elements, our work is the first to synthesize them into a comprehensive evaluation framework. Among all known risk factors, our research has specifically highlighted the issue of patients presenting with CT scans showing involvement in all five lobes of the lungs upon admission. This innovative discovery has significant value in determining the severity of patient conditions and guiding early intervention. We found a positive relationship between oxygen saturation and the degree of aeration in normal lung tissue. Although this relationship is not very strong, it is a unique finding of our research. Furthermore, our study has identified concurrent diabetes, lymphocyte decline, and elevated D-dimer levels as relatively independent risk factors leading to the severity of COVID-19.

Limitations of this study include: first, it is a single-center study with a relatively small sample size of COVID-19 cases, which may affect the efficiency of statistical analysis; second, lung volume may vary depending on the amount of air inhaled prior to CT scans, which is directly related to individual inhalation volumes and is a limitation of CT quantitative volume measurement; finally, the retrospective study design is subject to certain data omissions and lacks long-term follow-up of patients, warranting larger-scale prospective studies to validate these findings.

## Conclusions

The presence of diabetes, a decrease in lymphocyte count, an increase in D-dimer levels, involvement of five lung lobes, and a reduced volume of normally aerated lung tissue are risk factors for the progression of COVID-19 to severe cases. In CT lung volume quantification, there is a correlation between the volume and proportion of normally aerated lung tissue and blood oxygen saturation, whereas no correlation was observed with the volume and proportion of damaged lung tissue affected by the virus.

## Data Availability

Due to the First Affiliated Hospital of Gannan Medical University’s confidentiality agreement and proprietary data concerns, the data sets generated and analyzed during this study are not publicly available. However, they can be made available upon reasonable request. Interested parties should contact the corresponding author for data access.
